# A New Classification of Anterior Choroidal Artery Aneurysms and Its Clinical Application

**DOI:** 10.3389/fnagi.2021.596829

**Published:** 2021-03-15

**Authors:** Yu Duan, Xuanfeng Qin, Qinqzhu An, Yikui Liu, Jian Li, Gong Chen

**Affiliations:** ^1^Department of Neurosurgery, Huadong Hospital, Fudan University, Shanghai, China; ^2^Department of Neurosurgery, Huashan Hospital, Fudan University, Shanghai, China

**Keywords:** anterior choroidal artery aneurysm, classification, endovascular intervention therapy, risk, embolization

## Abstract

**Background and Purpose:** The aim of this study was to compare the different subtypes of anterior choroidal artery (AChoA) aneurysm based on a new classification and to analyze the risk factors according to individual endovascular treatment (EVT).

**Methods:** In the new classification, AChoA aneurysms are classified into independent type (I type) and dependent type (II type) based on the relationship between the AChoA and the aneurysm. II type aneurysms have three subtypes, IIa (neck), IIb (body), and IIc (direct). We retrospectively analyzed 52 cases of AChoA aneurysm treated in our center between 2015 to 2019. There were 13 (25.0%) I type aneurysms, 24 (46.2%) IIa aneurysms, 15 (28.8%) IIb aneurysms, and no IIc type; 28 cases had a subarachnoid hemorrhage. According to our preoperative EVT plan for the different subtypes: II type should achieve Raymond-Roy Occlusion Class 1 (RROC 1) where possible. To protect the AChoA, it is best to preserve the neck of the IIa type aneurysms (RROC 2), and RROC 3 is enough for IIb type.

**Results:** Ten asymptomatic cases with minimal aneurysms were treated conservatively. Of the other cases, 42 were treated with individualized EVT (26 with a simple coil, 6 with balloon-assisted coiling, 7 with stent-assisted coiling, and 3 by flow diverter. Different subtypes had different RROC (Z = 14.026, *P* = 0.001). IIb type aneurysms (χ^2^ = 7.54, *P* = 0.023) were one of the factors related to temporary or permanent AChoA injury during surgery. Overall, two patients (IIa = 1, IIb = 1) developed contralateral hemiparesis.

**Conclusions:** The new classification diagram clearly shows the features of all types of AChoA aneurysm and makes EVT planning more explicit. The II type (particularly IIb) was a potential risk factor for AChoA injury.

## Introduction

Anterior choroidal artery (AChoA) aneurysms are rare, accounting for approximately 2–5% of all intracranial aneurysms (Locksley et al., 1966; Kim et al., 2009; Aoki et al., 2016). Compared with clipping, endovascular treatment (EVT) is an established treatment option for intracranial aneurysms with shorter hospital stays and better recovery specially in the elderly people (Sadamasa et al., 2014). When dealing with such aneurysms, it is important to pack the aneurysm more densely to maintain AChoA patency, however, the delicate AChoA can be easily injured, resulting in serious complications (Friedman et al., 2001; Kim et al., 2008; Kang et al., 2009; Andre et al., 2018).

Over the last few decades, several different classifications for AChoA aneurysms have been suggested; however, they have usually been based on the surgeon's subjective feeling. Some are used for clipping (Friedman et al., 2001; Heros, 2010; Li et al., 2012) and some only for EVT (Kim et al., 2008; Kang et al., 2009; Senturk et al., 2009). No unified standard has been established and the treatment of these aneurysms is still challenging. In this study, we propose a new classification of AChoA aneurysms and provide a comprehensive analysis of clinical efficacy and the risk factors for EVT based on the new classification.

## Methods

### Clinical Data

From January 2015 to October 2019, 52 patients with a diagnosis of AChoA aneurysm were recruited in our single center. The patients' mean age was 53.8 ± 9.4 years, and there were 21 males and 31 females. Twenty-eight cases (53.8%) presented as subarachnoid hemorrhage (SAH). Of these, 15 cases were Hunt-Hess grade I, 10 cases were grade II, and 3 were grade III.

### The New Classification of AChoA Aneurysms

**Figure 1:**
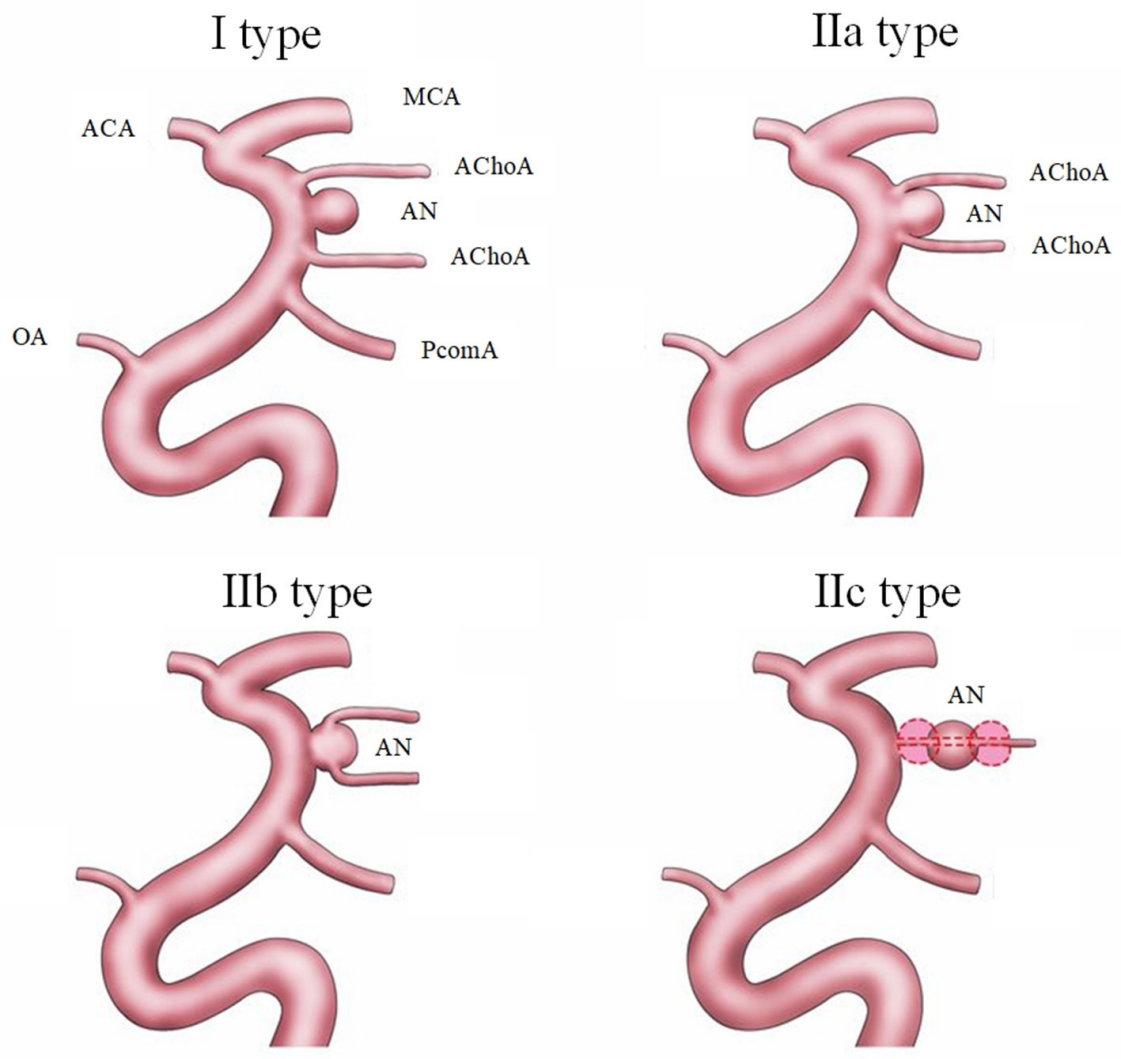
The new classification of AChoA aneurysms includes I type (dependent type) and II type (independent type). The II type is further divided into three subtypes: IIa type, (neck), where the AChoA originates from the neck of the aneurysm; the IIb type (body), where the AChoA originates from the body of the aneurysm; and the IIc type (direct), where the aneurysm originates directly from the AChoA.

The definitive diagnosis was made by digital subtraction angiography (DSA) and 3D-DSA in all patients. The aneurysms were divided into two types according to the relationship between the aneurysm and AChoA. The independent type (I type) and dependent type (II type) in the new classification are shown in Figure 1. In the I type, the aneurysm is located independently at the AChoA section of the internal carotid artery (ICA). In the II type, the aneurysm has a common trunk with the AChoA. In the IIa subtype the neck of the aneurysm is the origin of the AChoA (neck type), while in the IIb type the AChoA originates from the body of aneurysm (body type). The IIc type aneurysm is located directly at the AChoA (direct type). Of the 52 cases, 13 aneurysms (25.0%) were I type, 24 (46.2%) were IIa, 15 (28.8%) were IIb and there were no IIc type aneurysms (0%).

### Interventional Methods

First, having identified the subtype of aneurysm, the EVT target was determined. For I type aneurysms, as with other side-wall aneurysms, the aim was to reach Raymond-Ray Occlusion Class (RROC) 1 where possible. For II type aneurysms, however, it is more important to maintain AChoA patency; the neck must be preserved (RROC 2) for IIa, and RROC 3 is enough for IIb. For ruptured aneurysms, simple coiling and balloon-assisted coiling (BAC) would be the chosen method. Other features of the aneurysm, such as shape, neck width, height (neck-dome), height/width ratio, for example, were considerations for personalized EVT.

### Assessment and Follow Up

The degree of embolism, by RROC, was evaluated by DSA and CTA/MRA (Mascitelli et al., 2015). The clinical score was assessed by the modified Rankin Scale (mRS).

### Statistical Analysis

All data was analyzed by Social Science Version 25.0 for Windows (SPSS, Chicago, Illinois, USA) and presented as mean and SD for continuous variables or as number and percentage for categorical variables. Univariate analysis was performed using Pearson's χ^2^ (categorical variables) or one-way analysis of variance for three groups and independent samples *T* test for two groups (continuous variables, normal distribution), Mann–Whitney *U* test (abnormal distribution). *P* < 0.05 was taken as statistical significance.

## Results

### Operational Results

The ten asymptomatic cases (9.5%) with minor aneurysms (≤ 2.0 mm) were followed up without intervention. Of the 42 cases treated with EVT, the maximum aneurysm height varied from 1.5 mm to 5.5 (mean 3.51 ± 1.26 mm), the maximum neck width ranged from 1.5 to 5.3 mm (mean 3.26 ± 1.09 mm). Concerning shape, 81% (34/42) of aneurysms were regular and saccular, eight were irregular (fusiform, Gaussian-like shape, or biphasic shape). Multiple aneurysms occurred in 17 cases (40.5%, 17/42), while 13 (31%, 13/42) had a posterior communicating artery (PCA) aneurysm, 2 had an ophthalmic artery aneurysm, and 2 had a middle cerebral artery aneurysm. Thirty cases (71.4%, 30/42) were treated with simple coiling, 3 cases (7.9%, 3/42) with a flow diverter (FD-Tubridge, MicroPort Medical Company, Shanghai, China) and 9 cases (71.4%, 9/42) with stent-assisted coiling (SAC). States), and the other three cases, with Enterprise (Cordis Neurovascular, Miami Six of these were treated with Low-profile Visualized Intraluminal Support (MicroVention, Tustin, CA, United Lakes, FL, United States).

Based on the new classification, the different aneurysm types had different RROCs (Z = 14.046, *P* = 0.001, shown in Table 1). In addition, compared with unruptured aneurysms treated with SAC, ruptured aneurysms were embolized by coiling or BAC (Z = −2.833, *P* = 0.005, shown in Table 2). Five patients (four IIb type, one IIa type) suffered AChoA flow reduction or occlusion during the procedure and four were improved by adjusting the coils. The IIb type had the highest risk of AChoA injury (χ^2^= 7.54, *P* = 0.023). After EVT, two patients (all II type) presented with focal infarction of the AChoA area confirmed by CT or MRI, after showing AChoA-related symptoms and none of the AChoA aneurysm treated with flow diversion complicated with AChoA infarct.

**Table 1:** The characteristics of the different types.

	**I-type (*n* = 13)**	**IIa-type (*n* = 24)**	**IIb-type (*n* = 15)**	**Test value**	***P* value**
Female (*n*)	6	11	9	1.255^*^	0.534
SAH (*n*)	6	13	9	1.129^*^	0.569
Multiple (*n*)	4	8	5	1.105^*^	0.949
**EVT methods**					
Coiling	6	13	7	4.028^▵^	0.379
SAC	2	3	2		
BAC	1	1	1		
FD	1	1	1		
**RROC**				14.026^▵^	0.001
1	8	0	0		
2	3	17	0		
3a	0	2	10		
3b	0	0	2		
**Complications**					
AChoAOcclusion	0	1	4	7.54^*^	0.023
Infarction	0	1	1	0.898^*^	0.638
**DSA follow up**					
Improve	1	7	6		
Stable	9	10	5	1.54^▵^	0.215
Worsen	0	2	1		

* Pearson's χ^2^,

▵ *one-way analysis of variance*.

**Table 2:** The characteristics of ruptured and unruptured aneurysms.

	**Ruptured (*n* = 28)**	**Unruptured (*n* = 14)**	**Test value**	***P* value**
Female (*n*)	16	10	0.808^*^	0.368
Age (year)	55.39 + 10.48	50.79 + 7.60	1.46^▵^	0.152
Maximum height (mm)	3.61 + 1.26	3.31 + 0.65	0.853^▵^	0.399
Maximum height (mm)	3.20 + 0.99	3.38 + 0.56	−0.613^▵^	0.543
Multiple (*n*)	14	3	0.791^*^	0.374
**EVT methods**			−3.951^#^	0.00
Coiling	24	2		
SAC	0	7		
BAC	4	2		
FD	0	3		
**RROC**			−1.017^#^	0.309
1	4	4		
2	13	7		
3a	10	2		
3b	1	1		
**Complications**				
AChoA occlusion	4	1	0.875^*^	0.352
Infarction	1	1	0.263^*^	0.608
**DSA follow up**			−0.485^*^	0.628
Improve	9	6		
Stable	18	6		
Worsen	3	2		

* *Pearson's χ^2^*,

▵ *T test*,

# *Mann–Whitney U test*.

### Follow Up

All cases were followed up (from 7 to 62 months, mean 25 months) with assessment by MRA, CTA, or DSA. Three aneurysms (2 IIa, and 1 IIb) had degradation (two at the neck growth, and one at coil extrusion).

By the third postoperative month, two patients presented with progressive neurological improvement, with reductions in their mRS score from 5 to 3 and 3 to 1. During follow up, there were no recurrences of SAH, and no patients got worse or died. The AChoA aneurysms managed without EVT remained stable during follow up.

## Discussion

The terminal fields of supply of the AChoA are the posterior limb of the internal capsule, the beginning of the optic radiation, the lateral geniculate body, medial globus pallidus, and middle third of the crus cerebri (Rhoton et al., 1979; Champeaux et al., 2016; Ghali et al., 2018). If the AChoA is acutely occluded, it can lead to severe complications such as hemiplegia, hemianesthesia, homologous hemianopsia, and dysarthria, (called AChoA-syndromes), because of weak collateral circulation (Friedman et al., 2001). In 1968 and 1978, Drake and Yasargil, respectively, described the relationship between aneurysms and the AChoA according to their experience of a few cases treated by clipping (Drake et al., 1968; Yasargil et al., 1978). Because of technical limitations, the surgery treatment had not raised markedly at that period. In 2001, Friedman delineated three common anatomical variations of AChoA aneurysm according to the origins of AChoA and its spatial position in relation to the aneurysm (Friedman et al., 2001). In 2010, Heros classified the aneurysms into four types based on the spatial position of the aneurysm dome; these were the anterolateral, superolateral, posterolateral, and direct types (Heros, 2010). In 2012, Jin Li et al., summarized two groups based on the relationship between the AChoA and the aneurysm neck. In Group 1, the aneurysm originates from the ICA or the junction of the ICA and AChoA origin. In Group 2, the aneurysm originates entirely or in part from the AChoA itself. They found that Group 2 aneurysms had a higher risk of complications (Li et al., 2012). All the above classifications had significant differences and were based on the surgeons' subjective view. However, classifications based on spatial location does not assist EVT based on 3D-DSA.

Over the last decade, the use of EVT has gradually increased. The most common classification of aneurysms has two types (the independent type, and dependent type) (Kim et al., 2008; Kang et al., 2009; Senturk et al., 2009). In 2008, Cho added one more type, the direct type, in which the aneurysm originates entirely from the AChoA (Cho et al., 2008). In 2016, Aoki designated four types. In type A, the AChoA arises directly from the ICA, in type B, the AChoA arises from the aneurysmal neck, in type C, it arises from the aneurysmal dome, and in type D (truncal type) the aneurysms originate in a part of the AChoA itself. There are also several subtypes of B and C groups, according to the number of AChoA, which are too complicated for general classification criteria (Aoki et al., 2016). In our new classification, referring to the above classifications, we thought the first hierarchy must include the independent type (I type) and the dependent (II type). In the dependent type, it is important to differentiate the neck type (IIa), body type (IIb), and direct types (IIc). The new classification is clear and simple and could be applied for surgical clipping and EVT. We believe it will help to further establish a common, extensive, enforceable guideline.

**Figure 2:**
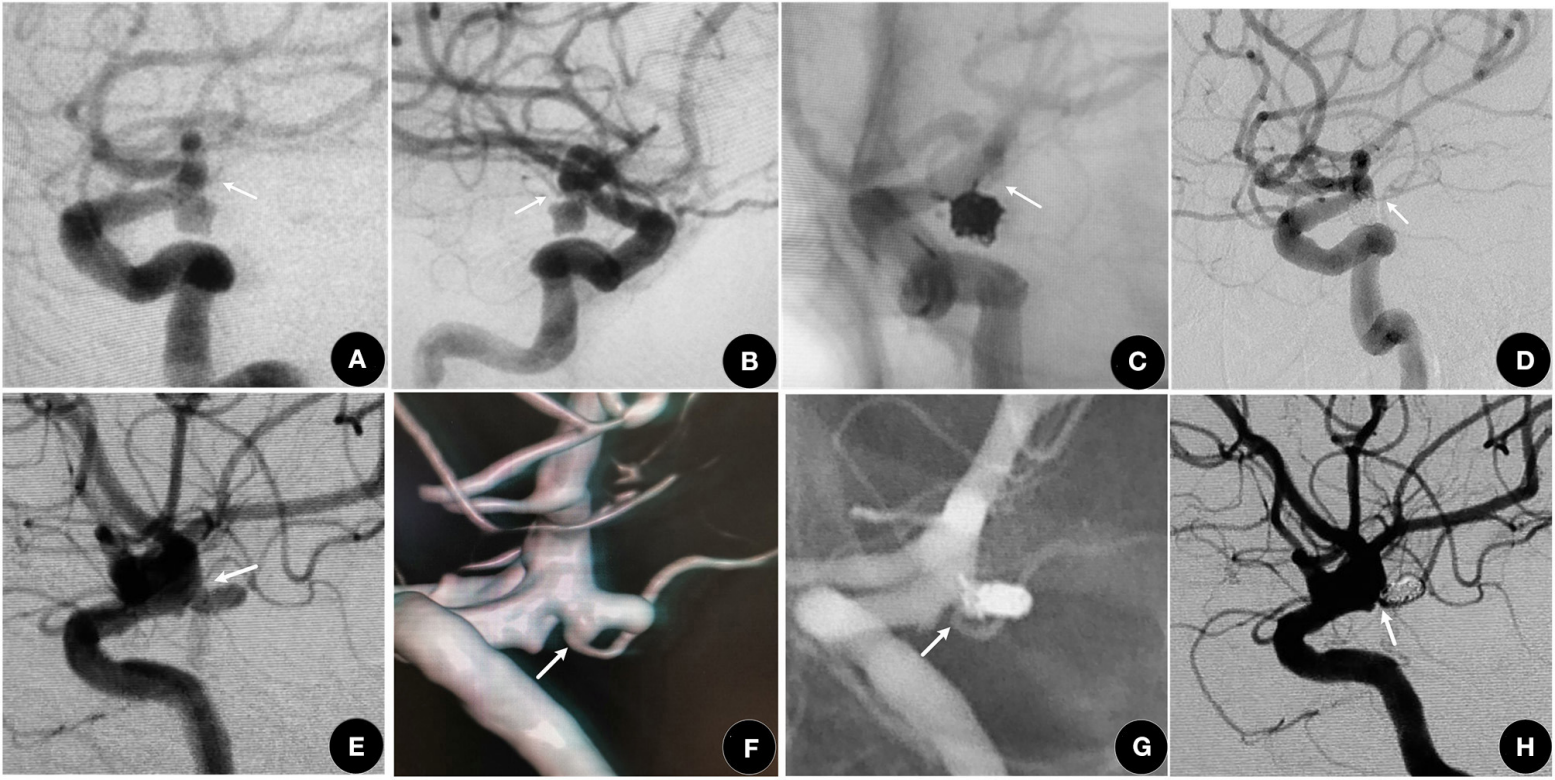
**(A–D)** A 63-year-old female who suffered with an SAH for 2 days. **(A,B)** showing a I type aneurysm. **(C)** RROC 1 was achieved using the double microcatheter technique and the AChoA (arrow) was well-protected. **(D)** One year later, the aneurysm was still at RROC 1 with a normal AChoA (arrow). **(E–H)** A 61-year-old female, suffered with SAH for 4 days. **(E,F)** showing a IIa type aneurysm (arrow). **(G,H)** The aneurysm was packed with the double microcatheter technique, reaching RROC 2. The AChoA (arrow) was well-protected.

**Figure 3:**
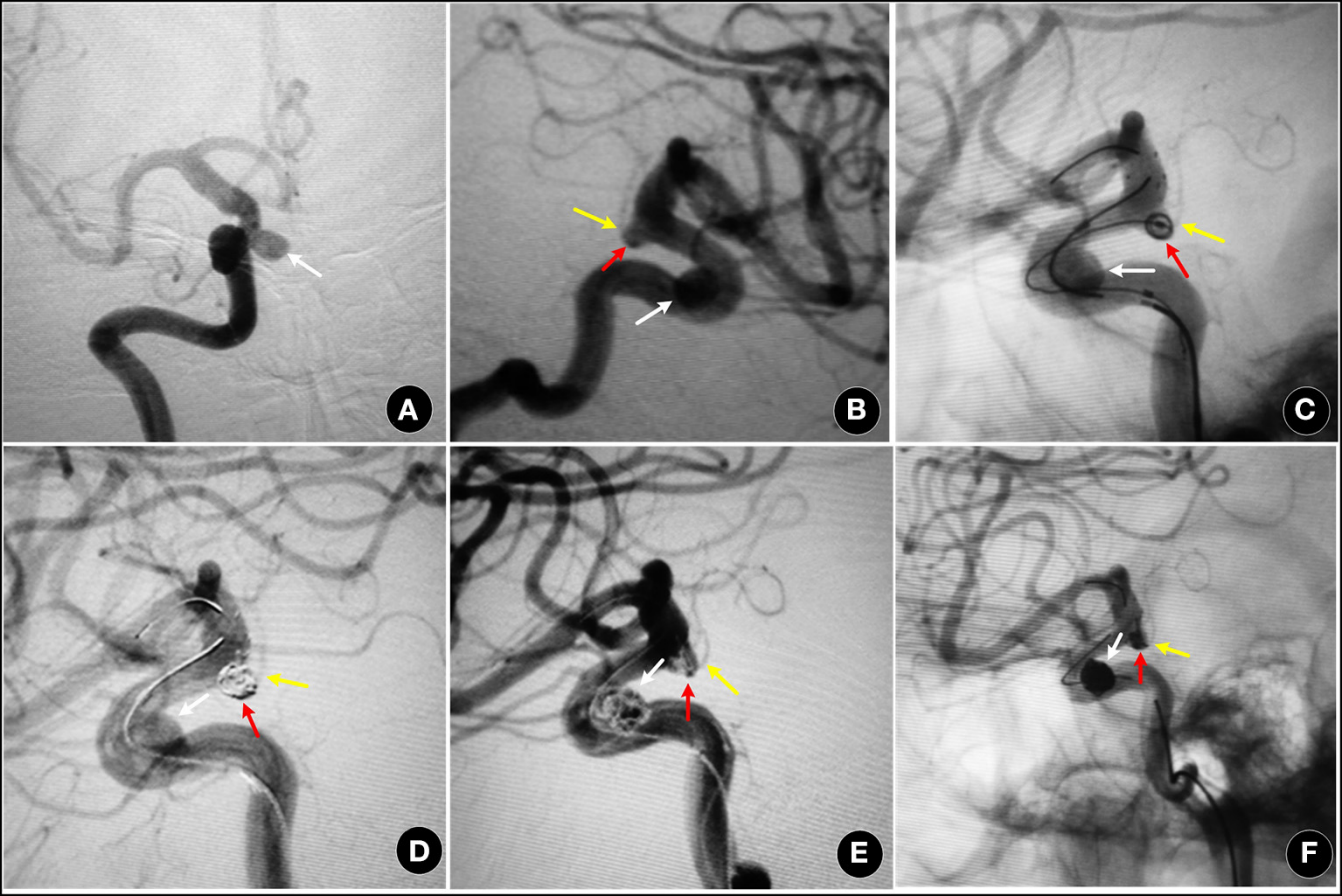
A 55-year-old female, with an aneurysm found on routine examination. **(A,B)** showing a IIb type aneurysm (red arrow) and an ipsilateral ophthalmic artery aneurysm (white arrow). **(C–F)** two aneurysms were treated by SAC (Enterprise stent), and partially packed (RROC 3a). The AChoA was well-preserved (yellow arrow).

According to the characteristics of the different subtypes, we redesigned the individual EVT plans. For I type, like the other side-wall aneurysms (Patel et al., 2014), 72.7% achieved complete obliteration without AChoA injury (shown in Figures 2A–D) and had a good clinical outcome (Andre et al., 2018). Based on protecting the AChoA, reaching RROC 2 in IIa type (shown in Figures 2E–H), and RROC 3a or 3b in IIb type were the goals (shown in Figure 3). To achieve the target, techniques such as double microcatheter, BAC, SAC or a combination of the above could be employed (Kim et al., 2010; Gimonet et al., 2016; Sheen and Suh, 2017). Even so, in this study, five II type (4 IIb, and 1 IIa) AChoA aneurysm cases suffered temporary or permanent injury, and two II type cases (4.8%, 2/42) developed the AChoA-syndrome. We found that II type, in particular IIb, had the highest risk (χ^2^ = 7.54, *P* = 0.023).

The IIc type aneurysms are extremely rare. We did not find this subtype in our study, which was a different result than Cho, who reported 24.5% (13/53) (Cho et al., 2008). We speculate that most of the IIc type aneurysms may be dissecting, fusiform, or dolichoectatic (Rutledge et al., 2019). The FD device may be a good choice for these, if there is no SAH.

**Figure 4:**
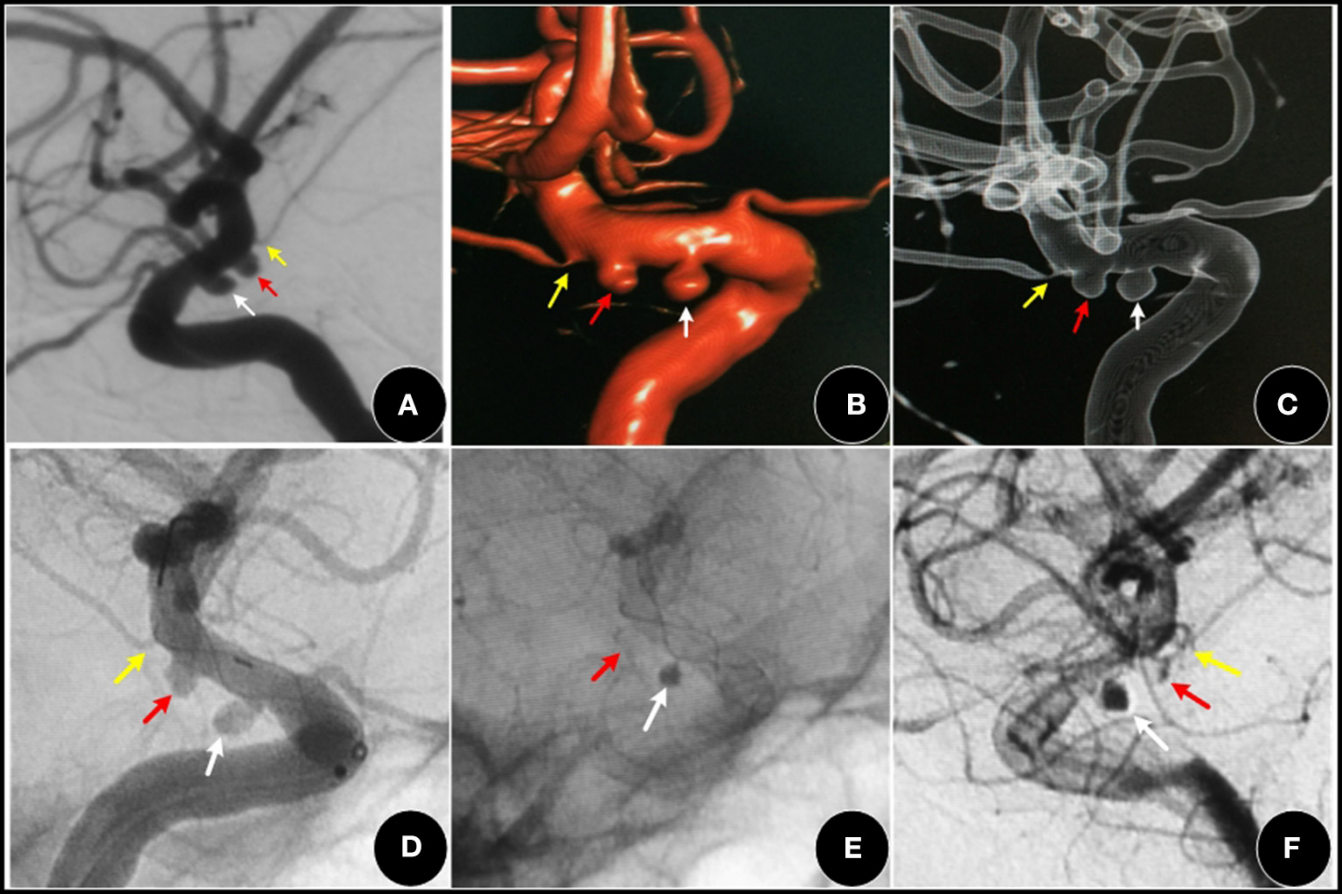
A 52-year-old female, with a three-month history of dizziness. **(A–C)** showing a I type aneurysm (red arrow) and an adjacent PCA aneurysm (white arrow). **(D–F)** two aneurysms were covered by an FD (Tubridge stent), the AChoA aneurysm was barely visible (red arrow). The PCA aneurysm (white arrow) showed obvious retention of contrast media, while the AChoA (yellow arrow) was patent.

It has been proven that the FD device is effective for aneurysm occlusion and the protection of perforated vessels in intracranial aneurysms. A multicenter experience showed that 83.3% of AChoA aneurysms achieved major occlusion and all AChoA were protected at follow up with the Pipeline embolization device (PED) (Srinivasan et al., 2018). Bhogal reported 30 cases of AChoA aneurysm treated by PED. Complete occlusion was reached in 50% of aneurysms immediately, and during follow up, 76.6% had also achieved it. All AChoAs were retained (Bhogal et al., 2019). In this study, three cases with a Turbridge stent in the ICA also achieved good results. The experience with FD devices in our center are as follows: the small II type aneurysms which were difficult to treat by conventional techniques (particularly the IIb and IIc type); those coexisting with adjacent multiple aneurysms (shown in Figure 4), and dissecting or fusiform aneurysms, and those without SAH.

At craniotomy, the AChoA may be more difficult to identify under the microscope, especially after SAH (Cho et al., 2008; Winkler et al., 2019), even with microvascular Doppler sonography (Shibata et al., 2000), intraoperative indocyanine green (Spiotta et al., 2011), or an intraoperative angiography device (Torne et al., 2019). SAH could lead to serious vasospasm and cerebral ischemia (Lee et al., 2018). In this cohort, four patients with SAH had AChoA injury, although this was not a statistically significant difference, SAH cannot be excluded as a risk factor. Based on the new classification, the long-term clinical effect of all treatment plans and relative risks need to be further researched.

Referring to the related literature (Senturk et al., 2009) and our 52 cases, the AChoA aneurysms have the following characteristics: more female patients (61.9%); a younger age (mean 53.9 years); a lower degree of SAH (84.6%, 25/28, Hunt-Hess I or II); a more regular shape (78.6%) and smaller size (41 cases ≤ 4 mm, 78.8%); more often associated with multiple aneurysms (32.7%, 17/52) (Senturk et al., 2009), especially with ipsilateral PCA aneurysm (76.5%, 13/17). As the number of cases and the length of follow up have increased, the features and outcomes of AChoA aneurysms will become clearer. There is another kind of AChoA aneurysm, the distal AChoA aneurysm. This is located at the distal part of the choroid point of the AChoA, and is rarely seen, except in conditions such as moyamoya disease (Choulakian et al., 2010). Because of its different pathogenic mechanism, lower risk, and treatment methods (Tanriover et al., 2014; Rutledge et al., 2019), this aneurysm is not included in the new classification. The new classification can also be called “AChoA originating aneurysms.”

### Study Limitations

The sample size here was small and follow-up was not long. In the future, these results based on new classification should be confirmed with a larger sample size and longitudinal follow up.

## Conclusions

The new classification diagram was simple and clear. We firstly and systematically differentiated the dependent type of AChoA aneurysm into IIa, IIb, and IIc type and summarized their features. The II type (specially IIb) was one of potential risk factors for AChoA injured during operation.

## Data Availability Statement

The raw data supporting the conclusions of this article will be made available by the authors, without undue reservation.

## Ethics Statement

The studies involving human participants were reviewed and approved by the ethics committee of Huashan hospital, Fudan university, Shanghai, China. The patients provided their written informed consent to participate in the study.

## Author Contributions

YD and GC: conceptualization, GC, XQ, QA, JL, and YD: surgery, YL and YD: data analysis, YD and GC: writing. All authors contributed to the article and approved the submitted version.

## Conflict of Interest

The authors declare that the research was conducted in the absence of any commercial or financial relationships that could be construed as a potential conflict of interest.
